# DBS emergency surgery for treatment of dystonic storm associated with rhabdomyolysis and acute colitis in DYT-GNAO1

**DOI:** 10.1007/s00381-022-05582-9

**Published:** 2022-06-20

**Authors:** Hind Chaib, Jan-Christoph Schoene-Bake, Assel Saryyeva, Thomas Jack, Hans Hartmann, Joachim K. Krauss

**Affiliations:** 1 Department of Neurosurgery, Hannover Medical School, Carl-Neuberg-Straße 1, 30625 Hannover, Germany; 2 Clinic of Pediatric Kidney, Liver and Metabolic Diseases, Hannover Medical School, Hannover, Germany; 3 Department of Human Genetics, Hannover Medical School, Hannover, Germany; 4 Department of Pediatric Cardiology and Pediatric Intensive Care, Hannover Medical School, Hannover, Germany

**Keywords:** Deep brain stimulation, Dystonic storm, DYT-GNAO1, Globus pallidus internus, GNAO1 gene

## Abstract

**Introduction:**

Patients with variants in the GNAO1 gene may present with life-threatening dystonic storm. There is little experience using pallidal deep brain stimulation (DBS) as an emergency treatment in such cases.

**Case description:**

We report on a 16-year-old girl with a variant in the GNAO1 gene (c.626G > T; p.(Arg209Leu)) who was admitted to the intensive care unit with medically refractory dystonic storm with secondary complications inducing rhabdomyolysis and acute colitis. Emergency pallidal DBS resulted in rapid improvement of dystonic storm and the subsidence of rhabdomyolysis and colitis. There were no further episodes of dystonic storm during follow-up of 2 years.

**Conclusion:**

Pallidal DBS is a useful treatment option for GNAO1-related dystonic storm with secondary complications which can be performed as an emergency surgery.

## Introduction

Dystonic storm can present as a life-threatening condition with a high risk of mortality and morbidity due to respiratory and metabolic complications [1–3]. The management of status dystonicus includes administration of high doses of sedatives, antispasmodic drugs, and treatment of triggering conditions such as infection [2, 4, 5]. In patients who do not respond well to medical treatment, both deep brain stimulation (DBS) and pallidal radiofrequency lesioning have been shown to provide rapid and effective relief [1, 3, 6, 7].

Variants in the GNAO1 gene (guanine-nucleotide-binding protein) have been identified in few patients with dystonic storm [7]. Such patients have been described to respond well to pallidal DBS [7–17]. Here, we report on the use of pallidal DBS as an emergency treatment in a 16-year-old girl with a variant in the GNAO1 gene who presented with a medically refractory dystonic storm complicated by severe rhabdomyolysis and acute colitis.

## Case report

This 16-year-old girl was admitted to the pediatric intensive care unit (ICU) with severe dystonic storm which had started after a tooth extraction under general anesthesia. She had a history of markedly delayed development since infancy. Seizures started during the first months of life, and she also developed episodes of varying intensity of dystonic movements of her arms and legs and later involving also the trunk. Initially, she had a diagnosis of cerebral palsy. She never learned to speak, but developed understanding of language, and she was later able to communicate with a talking device. She could not walk independently and was mobilized with a wheelchair and a walking frame. Further symptoms included hypersalivation and multiple contractures and luxations of her extremities which needed surgical correction.

**Fig. 1:**
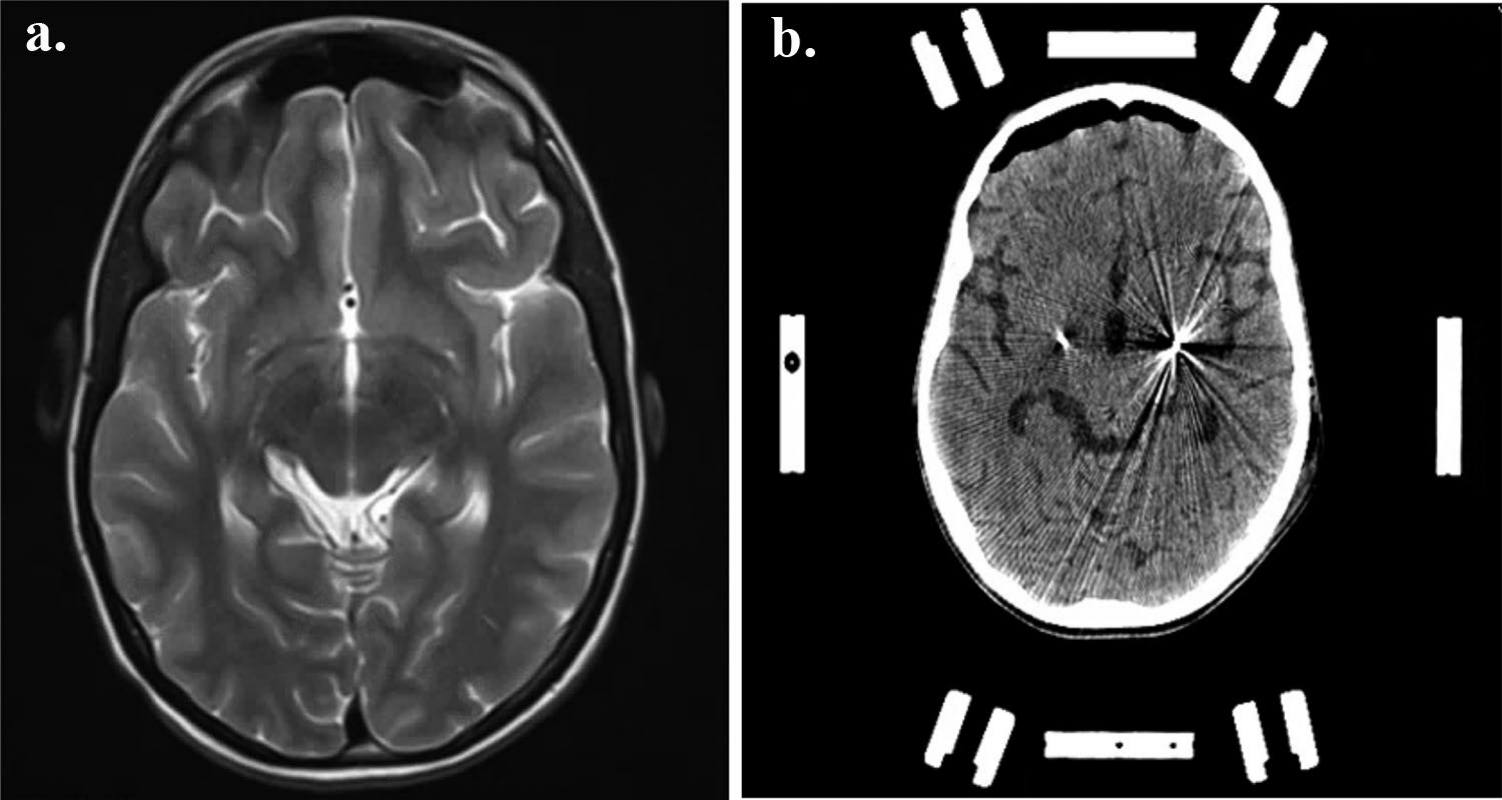
**a** MRI of the brain: T2-weighted axial image shows no abnormalities. **b** Postoperative stereotactic CT shows the positioning of the electrodes in the posteroventral lateral globus pallidus internus

Over the years, she had several episodes of dystonic storm, occasionally associated with rhabdomyolysis and renal impairment which were managed conservatively. Genetic analysis revealed a de novo heterozygous variant in the GNAO1 gene (c.626G > T; p.(Arg209Leu)). MR imaging of her brain was unremarkable (Fig. 1a).

After admission to the ICU at age 16, treatment was started with infusions of clonidine, midazolam, and morphine. Thereafter, continuous infusions of hydromorphone (6 µg/kg/h) and clonidine (1.2 µg/kg/h) were given which yielded little improvement of the dystonic storm. Additional treatment with clobazam, tetrabenazine, and gabapentin was ineffective.

Subsequently, hyperkalemia (6.9 mmol/l) and rhabdomyolysis (creatine kinase > 100.00 U/l) developed. Sedation with chloral hydrate resulted in improvement of dystonic storm; however, upon reduction of medication, dystonic storm reemerged over a period of 3 weeks. In the following, her state was further complicated by the development of acute colitis and pneumatosis hepatis.

With regard to her deteriorating condition, she was scheduled for emergency DBS surgery under general anesthesia. Quadripolar DBS electrodes (Vercise Cartesia Directional, Boston Scientific) were implanted bilaterally into the posteroventral lateral globus pallidus internus with CT stereotactic guidance supplemented by preoperative MR imaging and microelectrode recording as described in detail elsewhere [18, 19].

Subsequently, the electrodes were connected to an implantable pulse generator (Vercise PC, Boston Scientific). Postoperative stereotactic CT-imaging demonstrated appropriate electrode placement in the target bilaterally (Fig. 1b). Pallidal stimulation was started directly after completion of the surgery with the following settings: amplitude 2 mA, frequency 130 Hz, and triple monopolar electrode montage on both sides.

The dystonic movements improved rapidly after weaning from general anesthesia. On the second day after implantation of the electrodes, the medication with midazolam, clonidine, and hydromorphone could be reduced and was completely stopped over the next few days. The stimulation amplitude was increased in parallel to 2.9 mA. The rhabdomyolysis ceased, and the colitis subsided. The patient was discharged from the hospital 17 days after DBS surgery.

At 3-month follow-up, there was sustained marked improvement. No further episodes of dystonic storm had occurred. The patient could participate in daily activities again. She lived at home with her parents who were very satisfied with the result. At 2-year follow-up, she was in a stable condition.

## Discussion

Mutations in the GNAO1 gene were first identified in patients with epileptic encephalopathy. Subsequently, various phenotypes were defined characterized by severe developmental delay and hyperkinetic movement disorders [20]. GNOA1 is located on the long arm of chromosome 16 (16q12.2). It encodes the Gαo subunit of the guanine-nucleotide-binding protein, which has an important function in modulating transmembrane systems. Loss of function variants in the GNAO1 gene are associated with epilepsy, whereas gain of function variants are reported in patients with movement disorders [7, 14, 21]. In particular, disruption of the G-protein-cAMP pathway axis may be a key contributor to the pathophysiology of dystonia in these patients [7].

Pallidal DBS has become an accepted treatment option for various forms of dystonia [9, 22, 23]. In particular, inherited forms of dystonia tend to respond well to chronic pallidal stimulation [9]. While the benefit of DBS is often seen only with a delay of weeks and months in patients with dystonia [24], in some forms of dystonia, in particular in the case of dystonic storm, improvement may be observed within hours or days [9, 13, 15].

There is a limited experience with DBS in patients with GNAO1-related dystonia. According to the limited experience published thus far, these patients tend to respond well to pallidal DBS in the context of dystonic storm [7–17]. For instance, pallidal DBS has been used as an emergency measure to abate the severe dyskinesias and to restore normal daily functioning including feeding and sleeping [7, 13].

Our report highlights that pallidal DBS may be used as a life-saving treatment in an emergency context in patients with GNAO1-related dystonic storm abating also subsequent severe complications such as rhabdomyolysis and the rare occurrence of acute colitis.

## Data Availability

Upon reasonable request.
